# Extensions of the distributed lag non-linear model (DLNM) to account for cumulative mortality

**DOI:** 10.1007/s11356-021-13124-0

**Published:** 2021-03-18

**Authors:** Chao-Yu Guo, Xing-Yi Huang, Pei-Cheng Kuo, Yi-Hau Chen

**Affiliations:** 1 Institute of Public Health, School of Medicine, National Yang-Ming University, Taipei, Taiwan; 2 Institute of Public Health, School of Medicine, National Yang Ming Chiao Tung University, Hsinchu, Taiwan; 3 Department of Medicine, School of Medicine, National Yang-Ming University, Taipei, Taiwan; 4 Institute of Statistical Science, Academia Sinica, Taipei, Taiwan

**Keywords:** Distributed lag non-linear model, Multivariate analysis, Temperature, Mortality, Delayed effects

## Abstract

The effects of meteorological factors on health outcomes have gained popularity due to climate change, resulting in a general rise in temperature and abnormal climatic extremes. Instead of the conventional cross-sectional analysis that focuses on the association between a predictor and the single dependent variable, the distributed lag non-linear model (DLNM) has been widely adopted to examine the effect of multiple lag environmental factors health outcome. We propose several novel strategies to model mortality with the effects of distributed lag temperature measures and the delayed effect of mortality. Several attempts are derived by various statistical concepts, such as summation, autoregressive, principal component analysis, baseline adjustment, and modeling the offset in the DLNM. Five strategies are evaluated by simulation studies based on permutation techniques. The longitudinal climate and daily mortality data in Taipei, Taiwan, from 2012 to 2016 were implemented to generate the null distribution. According to simulation results, only one strategy, named MV_DLNM_, could yield valid type I errors, while the other four strategies demonstrated much more inflated type I errors. With a real-life application, the MV_DLNM_ that incorporates both the current and lag mortalities revealed a more significant association than the conventional model that only fits the current mortality. The results suggest that, in public health or environmental research, not only the exposure may post a delayed effect but also the outcome of interest could provide the lag association signals. The joint modeling of the lag exposure and the delayed outcome enhances the power to discover such a complex association structure. The new approach MV_DLNM_ models lag outcomes within 10 days and lag exposures up to 1 month and provide valid results.

**Supplementary Information:**

The online version contains supplementary material available at 10.1007/s11356-021-13124-0.

## Introduction

Extensive studies have indicated the association between temperature and human health, which arouses public health concerns as the climate has changed drastically on a worldwide scale due to global warming in recent years (Basu 2009; Gasparrini et al. 2010). After accounting for climate changes and other factors, how hot and cold weather, or their delayed effects, trigger human death were widely discussed in different areas, including the USA (Curriero et al. 2002; Mills et al. 2015), Europe (Baccini et al. 2008), and Northeast Asia (Chung et al. 2015). In addition to temperature, it has also been documented that exposure to air pollutants, which includes particulate matter (PM), ozone (O_3_), nitrogen dioxide (NO_2_), and sulfur dioxide (SO_2_) according to the 2005 WHO Air Quality Guidelines, leads to adverse effects on human health, especially the respiratory and cardiovascular diseases. Several types of research have examined the relationship between PM_10_, PM_2.5_, and daily mortality. Some showed that exposure to polluted air in a period would harm health conditions such as the development of lung or heart diseases, where the sources of pollution come from air, second-hand smoke, ozone, or particle matters (Dai et al. 2004; Janssen et al. 2013).

In 2010, Gasparrini et al. (2010) carried out the distributed lag non-linear model (DLNM) to evaluate predictors’ lag effect. The DLNM fits the non-linear association between the outcome variable and predictors. A cross-basis function simultaneously depicts the exposure–response relationship and the predictor space and lag–response relationship along with the lag space. In 2018, a new approach assessed both the same-day and 1-day lag mortality in DLNM (Chen et al. 2018). Therefore, associations in both lag outcomes and exposures need more attention to describe such a complex structure.

This research collected both weather and air pollution data as predictors and daily mortality as the health outcome in Taipei City from 2012 to 2016. Since the DLNM is widely adopted in public health and environmental research (Vicedo-Cabrera et al. 2016), we aim to extend the DLNM with Poisson link function and natural cubic splines (Bhaskaran et al. 2013) to model the cumulative mortality outcomes using lag predictors. The new methods’ validity and performance would be evaluated by the simulation study based on permutation techniques. Finally, a real data application shows a significant improvement attributable to the new method.

## Materials and methods

Anonymous daily mortality counts are the outcome of interest. Hence, no patients were involved in this research. All-cause mortality in Taipei City was obtained from the Cause of the Death Database published by the Ministry of Health and Welfare.

The Institutional Review Board (IRB) of National Yang-Ming University approved the use of anonymous mortality data (IRB number YM107045E). Daily mean temperature measures were obtained from Taipei Weather Station, available through the Central Weather Bureau (CWB n.d.) Observation Data Inquiry System website (CWB n.d.). Data on air pollution were obtained from Taipei Air Quality Monitoring System, available through Environmental Protection Administration Executive Yuan website (EPAEY n.d.), where we collected daily mean ozone concentration and daily mean PM_2.5_ concentration (Table 1). Although some air pollutions were missing, we could omit these observations since the missing rate is dismal, with an ignorable impact on the analyses.

**Table 1:** Daily all-cause deaths and three primary parameters, mean temperature, ozone concentration, and PM_2.5_ concentration, in Taipei from 2012 to 2016

**Daily data**	**Descriptive statistics**				
	A.1. *N*	Mean	SD	Min	Max
Mortality	1818	61.397	9.584	32	101
Mean temperature	1818	23.57	5.526	5.583	32.996
Mean *O*_3_	1816	28.030	9.628	6.317	81.967
Mean *PM*_2.5_	1801	20.459	10.413	3.714	87.143

The DLNM model is defined as the following:

$$ \log \left({\mu}_t\right)=\upalpha +\mathrm{s}\left({x}_t,l,\beta \right)+{\beta}_{O_3}{O_3}_t+{\beta}_{PM2.5} PM{2.5}_t+\kern0.5em \sum \limits_{i=1}^pf\left({z_t}^i;\theta \right) $$

The independent variable (*x*_*t*_) is daily mean temperature and other pollutant variables (*O*_3*t*_ and *PM*2.5_*t*_) are treated as potential confounders. The outcome variable (*μ*_*t*_) was all-cause mortality. The DLNM model was fitted through a cross-basis function s(*x*_*t*_, *l*, *β*) simultaneously describing the effect of the daily mean temperature *x*_*t*_ and its lag structure with maximum lag *l* on the expected mortality. Daily mean ozone concentration *O*_3*t*_ and daily mean concentration *PM*2.5_*t*_ are fixed effects. A natural cubic spline *f*(*z*_*t*_^*i*^ ; *θ*) with 8 degrees of freedom for each year is used to adjust for the seasonal effect. We selected 10, 20, and 30 days for the maximum exposure lag *l*. The cross-basis consists of a quadratic B-spline for temperature with the knots placed at 10th, 75th, and 90th percentiles and a natural cubic spline for the lag with 5 degrees of freedom, indicating three internal knots are equally spaced in the log scale.

In order to extend the DLNM to accommodate the lag mortalities, we propose five different multivariate (MV) approaches to transform the lag outcomes (*n* × *l*) into a one-dimensional dependent variable (*n* × 1) to be integrated by the DLNM.

For illustration purposes, assume that the Y matrix consists of 4 days of mortality with two lag days. Hence, the dimension of Y is (4 × 3). The second column of Y is the 1-day lag mortality. The third column of Y represents the 2-day lag mortality.

Let $$ Y=\left[\begin{array}{ccc}60& 51& 62\\ {}73& 60& 51\\ {}\begin{array}{c}61\\ {}55\end{array}& \begin{array}{c}73\\ {}61\end{array}& \begin{array}{c}60\\ {}73\end{array}\end{array}\right] $$, with $$ eigenvector=\left[\begin{array}{ccc}a& d& g\\ {}b& e& h\\ {}c& f& e\end{array}\right], eigenvalue=\left[\begin{array}{ccc}{\lambda}_1& 0& 0\\ {}0& {\lambda}_2& 0\\ {}0& 0& {\lambda}_3\end{array}\right] $$ in the principal component analysis (PCA).

Method 1:

MV_sum_: The most straightforward idea is to obtain the total mortalities from today to previous lag days. The new Y matrix (4 × 1) contains the summation of mortalities from the current day to the maximal lag day:

$$ {MV}_{sum}={\left[\begin{array}{c}60+51+62\\ {}73+60+51\\ {}61+73+60\\ {}55+61+73\end{array}\right]}_{4\ast 1}={\left[\begin{array}{c}173\\ {}184\\ {}194\\ {}189\end{array}\right]}_{4\ast 1} $$

Method 2:

MV_AR_: A commonly used longitudinal structure is autoregressive (AR). The lag mortality could be integrated into the current mortality by this weighted summation. The earlier a day lags, the less impact of mortality would contribute. We assumed a geometric progression with different ratios (0.8, 0.9, and 0.98):

i. The *n*-day lag mortality is multiplied by coefficients 0.8^*n*^
  $$ {MV}_{AR1}={\left[\begin{array}{ccc}60& 51& 62\\ {}73& 60& 51\\ {}\begin{array}{c}61\\ {}55\end{array}& \begin{array}{c}73\\ {}61\end{array}& \begin{array}{c}60\\ {}73\end{array}\end{array}\right]}_{4\ast 3}\ast {\left[\begin{array}{c}{0.8}^0\\ {}{0.8}^1\\ {}{0.8}^2\end{array}\right]}_{3\ast 1}={\left[\begin{array}{c}140.48\\ {}153.64\\ {}157.8\\ {}150.52\end{array}\right]}_{4\ast 1} $$
ii. The *n*-day lag mortality is multiplied by coefficients 0.9^*n*^
  $$ {MV}_{AR2}={\left[\begin{array}{ccc}60& 51& 62\\ {}73& 60& 51\\ {}\begin{array}{c}61\\ {}55\end{array}& \begin{array}{c}73\\ {}61\end{array}& \begin{array}{c}60\\ {}73\end{array}\end{array}\right]}_{4\ast 3}\ast {\left[\begin{array}{c}{0.9}^0\\ {}{0.9}^1\\ {}{0.9}^2\end{array}\right]}_{3\ast 1}={\left[\begin{array}{c}156.12\\ {}168.31\\ {}175.3\\ {}169.03\end{array}\right]}_{4\ast 1} $$
iii. The *n*-day lag mortality is multiplied by coefficients 0.98^*n*^
  $$ {MV}_{AR3}={\left[\begin{array}{ccc}60& 51& 62\\ {}73& 60& 51\\ {}\begin{array}{c}61\\ {}55\end{array}& \begin{array}{c}73\\ {}61\end{array}& \begin{array}{c}60\\ {}73\end{array}\end{array}\right]}_{4\ast 3}\ast {\left[\begin{array}{c}{0.98}^0\\ {}{0.98}^1\\ {}{0.98}^2\end{array}\right]}_{3\ast 1}={\left[\begin{array}{c}169.5249\\ {}180.7804\\ {}190.164\\ {}184.8892\end{array}\right]}_{4\ast 1} $$

Method 3:

MV_PCA_: The principal component analysis (PCA) (Jolliffe and Cadima 2016) is an unsupervised methodology to reduce numerous variables’ dimensionality. The first component represents the maximum variability explained. Therefore, we use only the first component in the first attempt. The second employs all eigenvectors such that all variabilities are maintained.

i. Only multiply the first eigenvector (to obtain the first principal component):
  $$ {MV}_{PCA1}={\left[\begin{array}{ccc}60& 51& 62\\ {}73& 60& 51\\ {}\begin{array}{c}61\\ {}55\end{array}& \begin{array}{c}73\\ {}61\end{array}& \begin{array}{c}60\\ {}73\end{array}\end{array}\right]}_{4\ast 3}\ast {\left[\begin{array}{c}a\\ {}b\\ {}c\end{array}\right]}_{3\ast 1}\to {\left[\begin{array}{c}60a+51b+62c\\ {}73a+60b+51c\\ {}61a+73b+60c\\ {}55a+61b+73c\end{array}\right]}_{4\ast 1} $$
ii. Multiply all eigenvector (to obtain all principal components) and the corresponding percentage:
  $$ {MV}_{PCA2}={\left[\begin{array}{ccc}60& 51& 62\\ {}73& 60& 51\\ {}\begin{array}{c}61\\ {}55\end{array}& \begin{array}{c}73\\ {}61\end{array}& \begin{array}{c}60\\ {}73\end{array}\end{array}\right]}_{4\ast 3}\ast {\left[\begin{array}{ccc}a& d& g\\ {}b& e& h\\ {}c& f& e\end{array}\right]}_{3\ast 3}\ast {\left[\begin{array}{c}\begin{array}{c}\frac{\lambda_1}{\left({\lambda}_1+{\lambda}_2+{\lambda}_3\right)}\\ {}\frac{\lambda_2}{\left({\lambda}_1+{\lambda}_2+{\lambda}_3\right)}\end{array}\\ {}\frac{\lambda_3}{\left({\lambda}_1+{\lambda}_2+{\lambda}_3\right)}\end{array}\right]}_{2\ast 1} $$

Method 4:

MV_adjust_: Separate the current mortality from the lag mortalities. Create a reduced matrix that sums over L lag mortalities but not the current mortality, $$ sum\left({x}_{sL}\right)={\left[\begin{array}{c}51+62\\ {}60+51\\ {}73+60\\ {}61+73\end{array}\right]}_{4\times 1}={\left[\begin{array}{c}113\\ {}111\\ {}133\\ {}134\end{array}\right]}_{4\times 1} $$, and adjust *sum*(*x*_*sL*_) as a covariate in the DLNM.

$$ {\left[\begin{array}{c}60\\ {}73\\ {}61\\ {}55\end{array}\right]}_{4\times 1}\mathrm{is}\ \mathrm{the}\ \mathrm{outcome}\kern0.5em \& sum\left({x}_{sL}\right)={\left[\begin{array}{c}113\\ {}111\\ {}133\\ {}134\end{array}\right]}_{4\times 1}\mathrm{is}\ \mathrm{adjusted}\ \mathrm{in}\ \mathrm{the}\ \mathrm{DLNM} $$

Method 5:

MV_DLNM_: Similar to method 4, but instead of treating the sum of previously lag mortalities as a covariate, $$ sum\left({x}_{sL}\right)={\left[\begin{array}{c}113\\ {}111\\ {}133\\ {}134\end{array}\right]}_{4\times 1} $$ is considered as the offset of the current mortality in the DLNM.

$$ {\left[\begin{array}{c}60\\ {}73\\ {}61\\ {}55\end{array}\right]}_{4\times 1}\mathrm{is}\ \mathrm{the}\ \mathrm{outcome}\kern0.5em \& sum\left({x}_{sL}\right)={\left[\begin{array}{c}113\\ {}111\\ {}133\\ {}134\end{array}\right]}_{4\times 1}\mathrm{is}\ \mathrm{the}\ \mathrm{offset}\ \mathrm{in}\ \mathrm{the}\ \mathrm{DLNM} $$

To validate the above approaches’ performance, we conducted a simulation study under the null hypothesis 1000 times. The null distribution was generated by permutations of mortality such that the outcome mortality and temperature measures were not correlated. The validity of each model was assessed. If the proportion of rejecting the null hypothesis does not exceed the significance level of 5%, the proposed strategy is a valid test.

All the statistical analyses and simulations were conducted by the software R (R Core Team (2014). R: A language and environment for statistical computing. R Foundation for Statistical Computing), equipped with the package "dlnm" by Gasparrini et al. (2010).

## Results

According to the permuted samples, the observed type I error for *MV*_*sum*_ is presented in Table 2. Methods 1 and 2 are based on the summation of previous outcomes but with different weights. Therefore, the results of *MV*_*AR*1_, *MV*_*AR*2_, and *MV*_*AR*3_ are similar and not shown.

**Table 2:** Type I errors of *MV*_*sum*_

	(10)	(20)	(30)
~Lag1	0.557	0.712	0.74
~Lag2	0.797	0.937	0.951
~Lag3	0.856	0.98	0.989
~Lag4	0.879	0.997	0.996
~Lag5	0.872	0.996	0.998
~Lag6	0.869	0.996	0.999
~Lag7	0.864	0.997	1
~Lag8	0.872	0.999	1
~Lag9	0.871	0.998	1
~Lag10	0.874	0.997	1
~Lag11		0.996	1
~Lag12		0.996	1
~Lag13		0.995	1
~Lag14		0.994	1
~Lag15		0.993	1
~Lag16		0.994	1
~Lag17		0.995	1
~Lag18		0.993	1
~Lag19		0.995	1
~Lag20		0.995	1
~Lag21			1
~Lag22			1
~Lag23			1
~Lag24			1
~Lag25			1
~Lag26			1
~Lag27			1
~Lag28			1
~Lag29			1
~Lag30			1

*MV*_*AR*1_, *MV*_*AR*2_, and *MV*_*AR*3_ are similar and not shown

Due to a negative value in principal components, *MV*_*PCA*1_ and *MV*_*PCA*2_ failed to satisfy the Poisson distribution model’s assumption and did not generate any DLNM package results. Hence, type I errors were not obtained. Note that type I error rates for *MV*_*sum*_, *MV*_*AR*1_, *MV*_*AR*2_, and *MV*_*AR*3_ were much larger than the nominal level of 0.05. Inflation is increasing for the number of lag outcomes. Therefore, these methods are not valid, although the idea is simple and could be easily implemented.

Table 3 shows that the type I error rate of MV_adjust_ was between 0.058 and 0.067 when the lag exposure was up to 10 days. The type I error became 0.083–0.183 when the lag exposures were up to 20 days. Finally, type I errors are 0.076–0.308 for 30 lag temperature measures. Although the type I error rate was consistently larger than 0.05, MV_adjust_ yielded much smaller type I errors than summation-based methods.

**Table 3:** Type I errors of *MV*_*adjust*_

	lag(10)	lag(20)	lag(30)
~Lag1	0.058	0.083	0.076
~Lag2	0.06	0.097	0.105
~Lag3	0.065	0.11	0.129
~Lag4	0.066	0.12	0.148
~Lag5	0.062	0.129	0.163
~Lag6	0.062	0.133	0.18
~Lag7	0.067	0.141	0.192
~Lag8	0.065	0.142	0.213
~Lag9	0.064	0.136	0.225
~Lag10	0.065	0.135	0.231
~Lag11		0.144	0.232
~Lag12		0.148	0.239
~Lag13		0.153	0.241
~Lag14		0.165	0.244
~Lag15		0.162	0.245
~Lag16		0.165	0.256
~Lag17		0.162	0.253
~Lag18		0.163	0.256
~Lag19		0.174	0.264
~Lag20		0.183	0.268
~Lag21			0.284
~Lag22			0.295
~Lag23			0.297
~Lag24			0.308
~Lag25			0.302
~Lag26			0.299
~Lag27			0.294
~Lag28			0.301
~Lag29			0.299
~Lag30			0.295

Finally, Table 4 shows that the type I error rate of MV_DLNM_ was smaller than 0.05 when the lag exposure was 10 days. The type I error would range from 0 to 0.078 if the lag exposure were 20 days. When the lag exposure was 30 days, the type I error ranges from 0 to 0.102. Therefore, the results indicated that MV_DLNM_ is the only valid test. For the 10, 20, and 30 lag exposures, the cumulative outcome mortality could be implemented up to 10, 10, and 13 days, respectively.

**Table 4:** Type I errors of *MV*_*DLNM*_

Mortality	lag(10)	lag(20)	lag(30)
~Lag1	0	0	0
~Lag2	0.001	0	0
~Lag3	0.008	0	0
~Lag4	0.026	0.001	0
~Lag5	0.03	0.004	0
~Lag6	0.034	0.009	0.002
~Lag7	0.035	0.019	0.002
~Lag8	0.036	0.028	0.005
~Lag9	0.043	0.031	0.009
~Lag10	0.049	0.04	0.014
~Lag11		0.052*	0.02
~Lag12		0.055*	0.029
~Lag13		0.052*	0.038
~Lag14		0.056*	0.056*
~Lag15		0.058*	0.065*
~Lag16		0.064*	0.072*
~Lag17		0.068*	0.079*
~Lag18		0.073*	0.087*
~Lag19		0.074*	0.089*
~Lag20		0.078*	0.087*
~Lag21			0.085*
~Lag22			0.086*
~Lag23			0.091*
~Lag24			0.092*
~Lag25			0.091*
~Lag26			0.098*
~Lag27			0.099*
~Lag28			0.099*
~Lag29			0.098*
~Lag30			0.102*

*These *p* values indicate the inflated type I errors

The research aims to provide a novel method with an ensured valid type I error and sufficient statistical power. Therefore, in addition to type I error simulations, we examined computer simulations to compare the performance between the DLNM and the MV_DLNM_. We conducted 1000 repetitions for each scenario. The R function for power simulation is freely available. Researchers could use various datasets with different environmental factors and structures in other countries worldwide to confirm that both the lag outcomes and exposures could demonstrate a significant association.

In power simulations, we kept the temperature and air pollution structures in Taipei from 2012 to 2016. According to the Poisson distribution, we simulated the outcome variable with the mean parameter λ equals the daily mean temperature. In this way, the temperature determines the number of mortality, and the association is significant. Scenarios included different lengths of the study period from 120 days to 1 year since the statistical power is 100% for both methods with a sample size of more than 1 year. Therefore, besides statistical power, we recorded the percentage when the *p* value of the MV_DLNM_ is smaller than the *p* value of the DLNM. Simulation results revealed that the MV_DLNM_ outperforms the conventional DLNM in the scenarios we examined (Table 5). The percentage when the MV_DLNM_ reveals a more significant result is higher than 50%, and the power of the MV_DLNM_ is consistently higher than the power of the DLNM.

**Table 5:** Power simulations with 1000 repetitions

Study period	Lag exposure	Power MV_DLNM_	Power DLNM	MV_DLNM_ Wins
1 year	10	0.999	0.999	0.7640
240 days	10	0.996	0.993	0.88
160 days	10	0.928	0.893	0.835
120 days	10	0.691	0.618	0.781
1 year	20	1	0.999	0.787
240 days	20	0.995	0.992	0.917
160 days	20	0.898	0.861	0.883
120 days	20	0.651	0.531	0.906
1 year	30	1	0.999	0.764
240 days	30	0.996	0.986	0.878
160 days	30	0.87	0.833	0.822
120 days	30	0.636	0.516	0.897

The column “MV_DLNM_ Wins” represents the percentage when the *p* value of the MV_DLNM_ is smaller than the *p* value of the DLNM

**Fig. 1:**
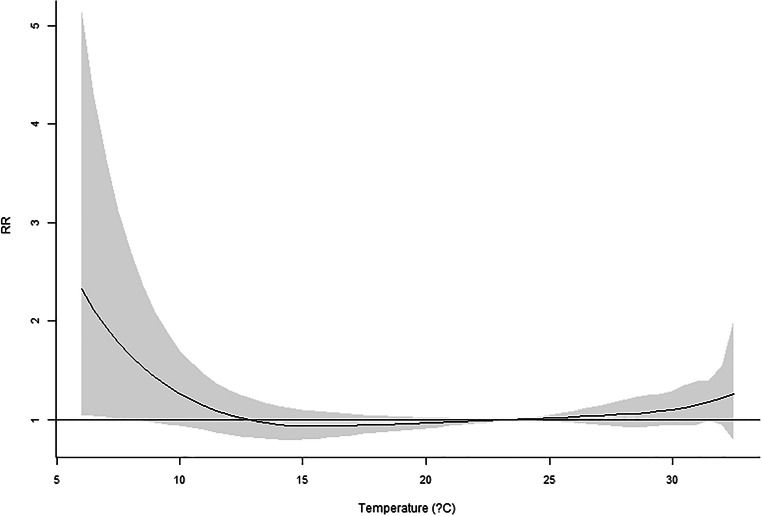
Overall RR on the current day

**Fig. 2:**
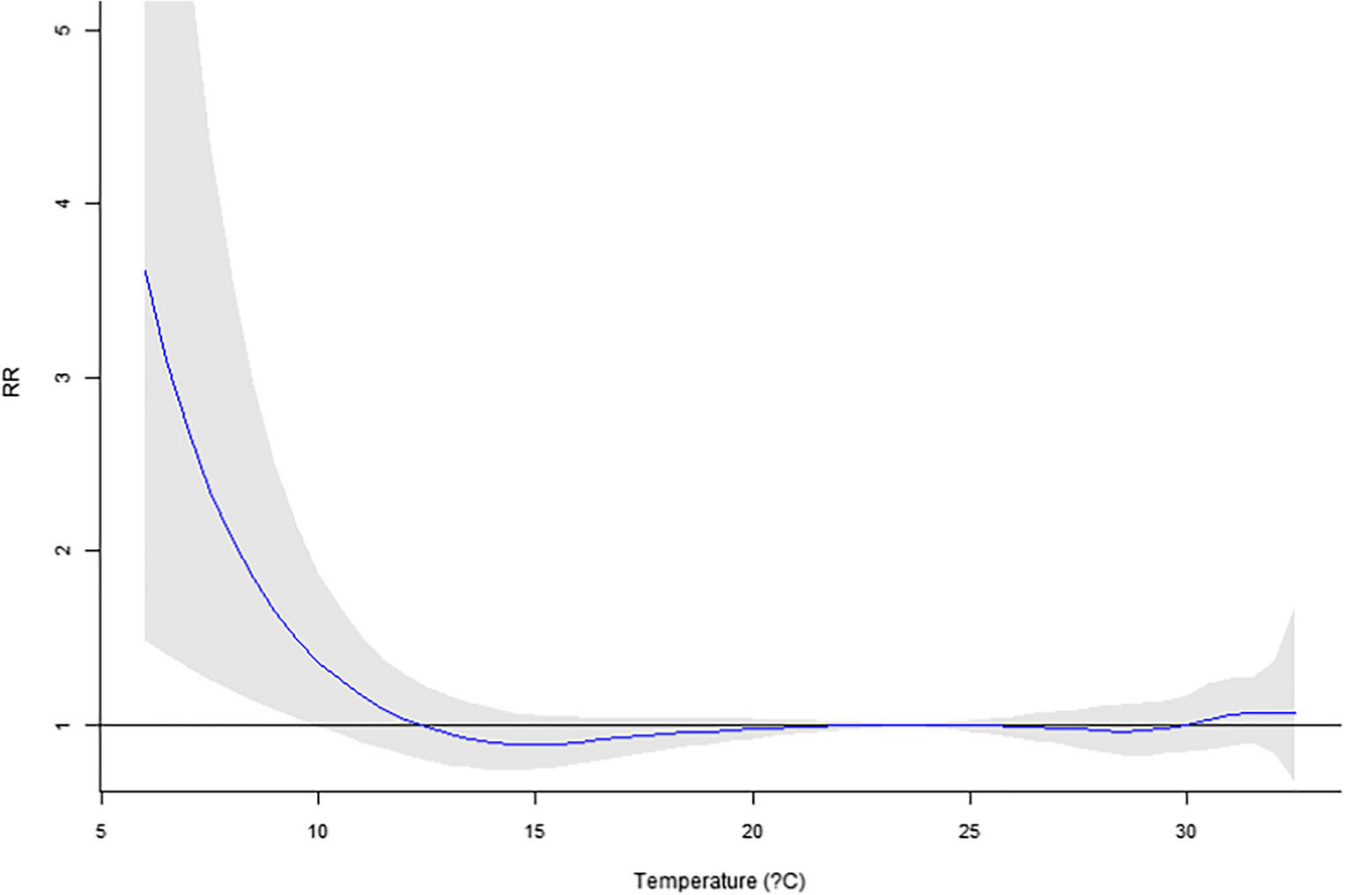
Overall RR on the 5th lag day

**Fig. 3:**
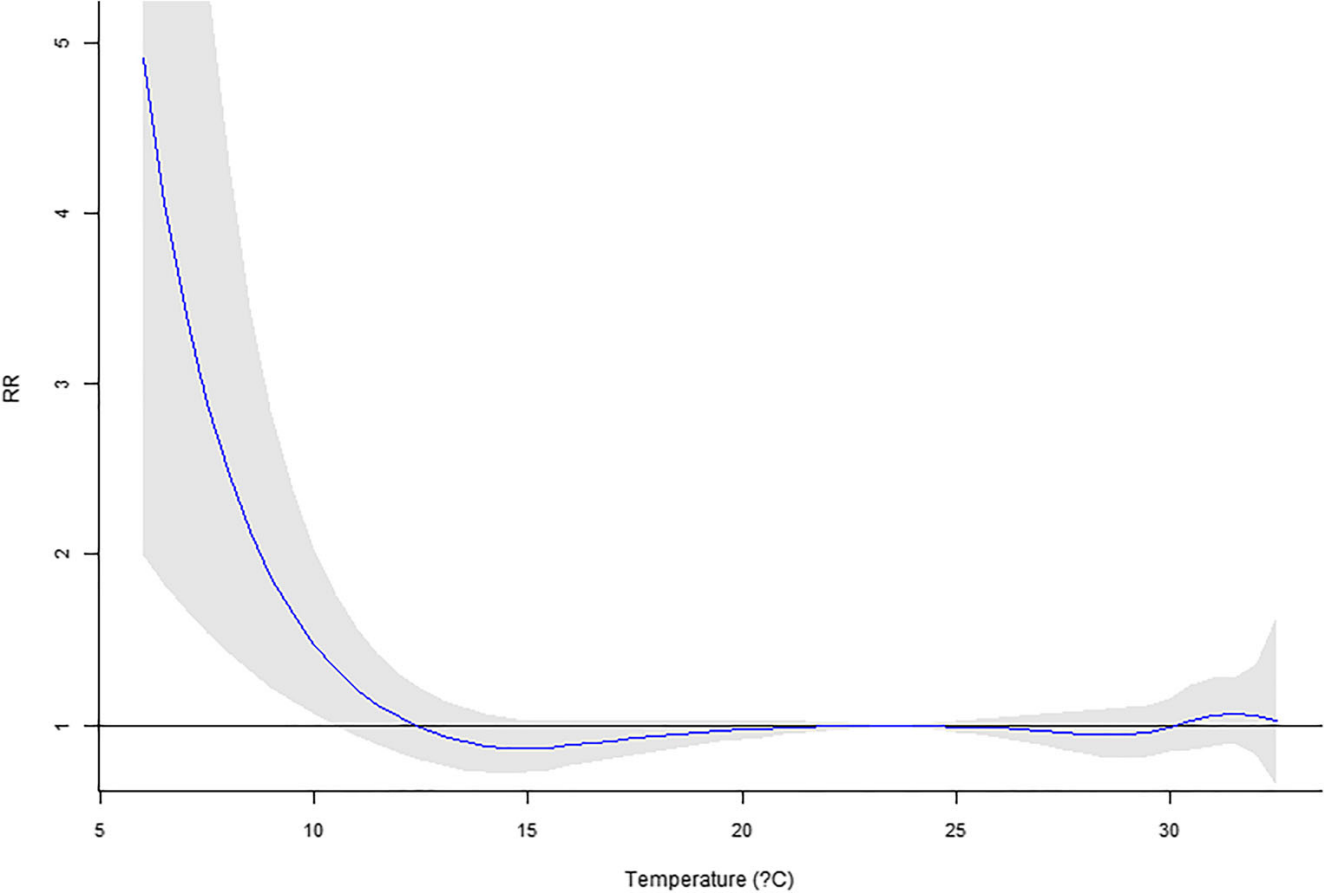
Overall RR on the 10th lag day

**Fig. 4:**
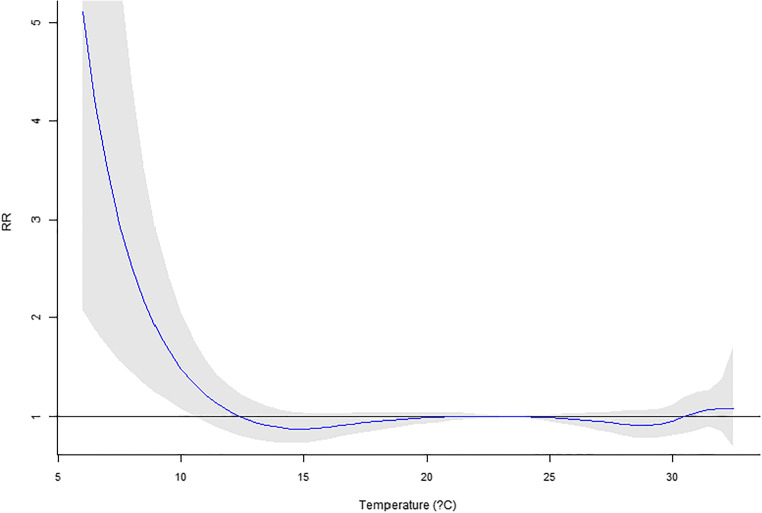
Overall RR on the 20th lag day

**Fig. 5:**
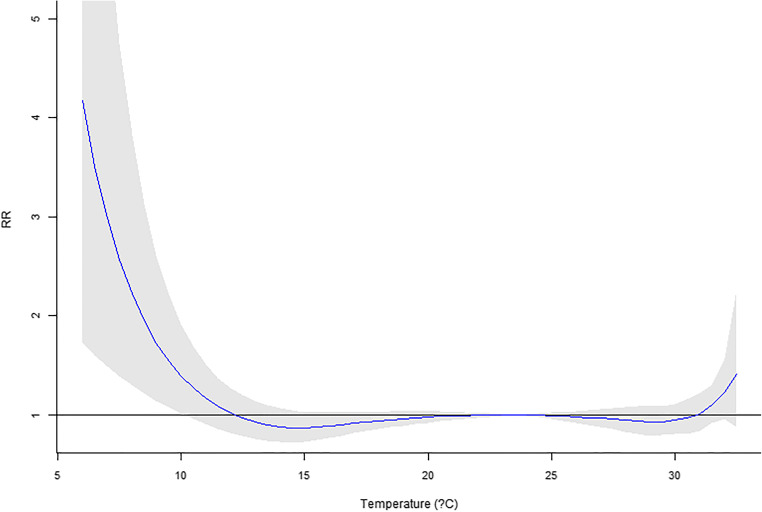
Overall RR on the 30th lag day

Our previous work using six major cities in Taiwan (Guo et al. 2014) reported a significant temperature impact on mortality. In this research, only Taipei City is available for recent years. However, the MV_DLNM_ could provide significant overall *p* values (Table 6). Regarding the temperature measure up to 10 lag days, significant *p* values were observed for four or more lag mortalities incorporated in the model. For temperature in 20 and 30 lag days, if five or more lag mortalities are used in the MV_DLNM_, the result would suggest significant associations. Hence, the cumulative outcomes could contribute to the association with lag exposures. In Figs. 1, 2, 3, 4, and 5, the overall relative risk (RR) for 30 lag days is displayed. The RR on the current day is approximately 2.3, but the RR increases with respect to the lag effects. For the lag of 10 days, the RR is as high as 5. The figures on the rest lag days were not shown. There are too many figures, and the pattern was observed according to the five figures.

**Table 6:** Real data application of MV_DLNM_: overall *p* value

	lag(10)	lag(20)	lag(30)
~Lag1	0.807516	0.996482	0.999951
~Lag2	0.100355	0.489865	0.945508
~Lag3	0.009709	0.045561	0.338491
~Lag4	0.001388^#^	0.009588	0.060874
~Lag5	0.000231^#^	0.001842^#^	0.012078^#^
~Lag6	0.000107^#^	0.000605^#^	0.004511^#^
~Lag7	0.000120^#^	0.000350^#^	0.001656^#^
~Lag8	0.000108^#^	0.000198^#^	0.000551^#^
~Lag9	0.000076^#^	0.000088^#^	0.000184^#^
~Lag10	0.000031^#^	0.000019^#^	0.000045^#^
~Lag11		0.000009*	0.000017^#^
~Lag12		0.000009*	0.000010^#^
~Lag13		0.000014*	0.000011^#^
~Lag14		0.000020*	0.000015*
~Lag15		0.000019*	0.000015*
~Lag16		0.000009*	0.000010*
~Lag17		0.000003*	0.000005*
~Lag18		0.000002*	0.000003*
~Lag19		0.000001*	0.000001*
~Lag20		0.000001*	0.000001*
~Lag21			0.000001*
~Lag22			0.000001*
~Lag23			0.000002*
~Lag24			0.000003*
~Lag25			0.000004*
~Lag26			0.000006*
~Lag27			0.000007*
~Lag28			0.000009*
~Lag29			0.000014*
~Lag30			0.000021*

^#^Denotes significant *p* values

*Denotes invalid type I error rates indicated by the permutation study in Table 4

Since the MV_DLNM_ extends the DLNM with an offset, the MV_DLNM_ models the mortality rates comparing to the DLNM that models mortality counts. The cross-basis and all covariate structures of the MV_DLNM_ are identical to that of the DLNM. This phenomenon is also an advantage of the new approach.

## Discussion

Conventional DLNMs can be interpreted as one day’s exposure influences outcome over several subsequent days, discussed in various publications by Gasparrini. However, the DLNM only considers the outcome on the current day. In this research, several strategies were proposed to explore further the possibilities of extending the DLNM to incorporate the previous days’ outcomes. Looking at the specific models proposed, one would think that what this research means is that mortality on one day depends on mortality on several previous days (not just exposures). Most statisticians would consider these as autoregression models. Therefore, this research also provides epidemiological motivation, noting the potential reasons for such autocorrelation, which may be introduced by unmeasured slow-changing covariates, such as infectious diseases (Imai et al. 2015). Simple autoregression (Brumback et al. 2000) has been considered in the environmental time series literature, but not much discussion of the various types of models proposed here (though there is some—e.g., Imai et al. 2015).

In a different point of view, all of the models proposed in this research could be considered as DLNMs for the dependence of mortality on earlier mortality: (1) MV_sum_ is equivalent to stratum-constrained DLNM; (2) MV_PCA_ is the same as DLNM with lag weights determined by PCA; (3) MV_adjust_ is a different stratum-constrained DLM; (4) MV_DLNM_ could be considered as MV_adjust_ but with coefficient constrained to 1.

Through simulation studies, we examined several novel approaches to characterize the effect of the delayed mortality and lag temperature measures. Results suggested that most methods are invalid, although these statistical concepts are intuitive and could be implemented effortlessly. The negative findings could provide researchers a great idea to avoid such types of analyses. Fortunately, there is one valid model, the MV_DLNM_, where the log-transformed summation of the delayed mortalities is treated as an offset in the DLNM model. The MV_DLNM_ model is $\log \left({\mu}_t\right)=\upalpha +\mathrm{s}\left({x}_t,l,\beta \right)+{\beta}_{O_3}{O_3}_t+{\beta}_{PM2.5} PM{2.5}_t+\kern0.5em {\sum}_{i=1}^pf\left({z_t}^i;\theta \right)+ offset\left(\log \left( sum\left({x}_{sL}\right)\right)\right)$.

Because the new method MV_DLNM_ could not be easily implemented in the DLNM package, we provide the R functions for researchers to utilize the MV_DLNM_ effortlessly. The example data in Taipei City is also enclosed. Please see the supplementary materials for details. The R code generates the plots for relative risks. Besides, we prepared another R function for power simulations such that researchers could assess if their environmental data have the advantage of incorporating the lag outcomes in addition to the lag exposures. We have made the corresponding changes accordingly.

The illustration of real data analysis of Taipei City from 2012 to 2016 confirmed that the delayed mortality records could significantly increase the association signal along with lag temperature measures, which matches the conclusions as we previously reported (Guo et al. 2014). Nevertheless, this new strategy is a handy tool and could be adopted by various research fields when the cumulative outcome provides a more significant signal than the current one.

In public health research, the exposure may post a delayed effect, but the outcome of interest could signal the lag effect. This methodological study provides a simple yet valid test that jointly models the lag exposure and the delayed mortality records to enhance the ability to discover such a complex association structure.

In summary, this research proposed a novel strategy to account for cumulative mortality in the distributed lag temperature records. According to computer simulations, the new model MVDLNM demonstrated a much more significant association than the conventional method with the current mortality.

The simulation results revealed that the type I error of MV_DLNM_ does not exceed the nominal level of 5% within ten lag mortalities. Since 10 days is an intuitive interval, we recommend incorporating up to 10 days of lag outcomes in the new approach. In conclusion, the new approach MV_DLNM_ models lag outcomes within 10 days and lag exposures up to 1 month and provide valid results.

## Strengths and limitations

This research proposed several novel statistical models accounting for daily mortality in previous days. Although the concept is intuitive and one could quickly implement the methods, the four methods were not valid tests. However, the negative findings could prevent researchers from such types of erroneous models. Comparing to the conventional analysis model that only assesses the current mortality, the new approach MV_DLNM_ yielded a much more significant association. The RR’s maximum value in Fig. 1 increases to the RR in Fig. 5 and showed explicit evidence that the lag exposure and outcome of interests contribute to the statistical significance.

The data used in this study are limited to Taipei, the capital of Taiwan, while the relationship between temperature and mortality may consist of various profiles in other regions. For example, the accessibility and quality of medical care may be different in smaller towns. In addition, we considered the all-cause mortality since we could not further classify death causes into more categories, such as sudden cardiac death or myocardial infarction, which are more likely to be related to temperature and air pollution. As for the temperature, only daily mean temperature was considered in this study. We did not explore the highest, lowest temperature, and intraday temperature variation in the contribution to human death. Finally, some researchers proposed a threshold to differentiate the impact of hot and cold temperatures on mortality. In contrast, we use the continuous temperature measures to employ spline functions and polynomials.

## Supplementary Information

ESM 1 (R 3 kb)

ESM 2 (DOCX 18 kb)

ESM 3 (DOCX 18 kb)

ESM 4 (R 5 kb)

ESM 5 (CSV 128 kb)

## References

[CR1] Baccini M, Biggeri A, Accetta G, Kosatsky T, Katsouyanni K, Analitis A, Anderson HR, Bisanti L, D'Ippoliti D, Danova J, Forsberg B, Medina S, Paldy A, Rabczenko D, Schindler C, Michelozzi P (2008). Heat effects on mortality in 15 European cities. Epidemiology.

[CR2] Basu R (2009). High ambient temperature and mortality: a review of epidemiologic studies from 2001 to 2008. Environ Health.

[CR3] Bhaskaran K, Gasparrini A, Hajat S, Smeeth L, Armstrong B (2013). Time series regression studies in environmental epidemiology. Int J Epidemiol.

[CR4] Brumback BA, Ryan LM, Schwartz JD, Neas LM, Stark PC, Burge HA (2000). Transitional regression models, with application to environmental time series. J Am Stat Assoc.

[CR5] Chen C-C, Lin B-C, Yap L, Chiang P-H, Chan T-C (2018). The association between ambient temperature and acute diarrhea incidence in Hong Kong, Taiwan, and Japan. Sustainability.

[CR6] Chung Y, Lim YH, Honda Y, Guo YLL, Hashizume M, Bell ML, Chen BY, Kim H (2015). Mortality related to extreme temperature for 15 cities in northeast Asia. Epidemiology.

[CR7] Curriero FC, Heiner KS, Samet JM, Zeger SL, Strug L, Patz JA (2002). Temperature and mortality in 11 cities of the eastern United States. Am J Epidemiol.

[CR8] CWB (n.d.) https://eservice.cwb.gov.tw/HistoryDataQuery/index.jsp. Accessed 6 Sept 2018

[CR9] Dai H, Song W, Gao X, Chen L (2004). Study on relationship between ambient PM10, PM2. 5 pollution and daily mortality in a district in Shanghai. Wei sheng yan jiu=. J Hygiene Res.

[CR10] EPAEY (n.d.) Environmental Protection Administration Executiv Yuan. https://erdb.epa.gov.tw/DataRepository/EnvMonitor/AirQualityMonitorDayData.aspx?topic1=%E5%A4%A7%E6%B0%A3&topic2=%E7%92%B0%E5%A2%83%E5%8F%8A%E7%94%9F%E6%85%8B%E7%9B%A3%E6%B8%AC&subject=%E7%A9%BA%E6%B0%A3%E5%93%81%E8%B3%AA. Accessed 6 Sept 2018

[CR11] Gasparrini A, Armstrong B, Kenward MG (2010). Distributed lag non-linear models. Stat Med.

[CR12] Guo CY, Pan WC, Chen MJ, Tsai CW, Chen NT, Su HJ (2014). When are we most vulnerable to temperature variations in a day?. PLoS One.

[CR13] Imai N, Dorigatti I, Cauchemez S, Ferguson NM (2015). Estimating dengue transmission intensity from sero-prevalence surveys in multiple countries. PLoS Negl Trop Dis.

[CR14] Janssen N, Fischer P, Marra M, Ameling C, Cassee F (2013). Short-term effects of PM2. 5, PM10 and PM2.5–10 on daily mortality in the Netherlands. Sci Total Environ.

[CR15] Jolliffe IT, Cadima J (2016). Principal component analysis: a review and recent developments. Philos Transact Series A Math Phys Eng Sci.

[CR16] Mills D, Schwartz J, Lee M, Sarofim M, Jones R, Lawson M, Duckworth M, Deck L (2015). Climate change impacts on extreme temperature mortality in select metropolitan areas in the United States. Clim Chang.

[CR17] R Core Team (2014) R: A language and environment for statistical computing. R Foundation for Statistical Computing V, Austria. http://www.R-project.org/. Accessed 6 Sept 2018

[CR18] Vicedo-Cabrera AM, Forsberg B, Tobias A, Zanobetti A, Schwartz J, Armstrong B, Gasparrini A (2016). Associations of inter- and intraday temperature change with mortality. Am J Epidemiol.

