# Supplementary material for: Extensions of the distributed lag non-linear model (DLNM) to account for cumulative mortality
Source: Environ Sci Pollut Res Int. 2021 Mar 18;28(29):38679–88. doi: 10.1007/s11356-021-13124-0 (PMC8310484; doi:10.1007/s11356-021-13124-0)
Supplement: Supplementary file 2 — (DOCX 18 kb) [file 11356_2021_13124_MOESM2_ESM.docx]

**‘MV.DLNM’**

**Type** Function

**Title** Extensions of the Distributed Lag Non-Linear Model (DLNM) to Account for Cumulative Mortality

**Version** 1.0

**Date** Feb 17 2021

**Author** Chao-Yu Guo, Ph.D., Xing-Yi Huang, MS, Pei-Cheng Kuo, MD, Yi-Hau Chen, Ph.D.

**Contact** Dr. Chao-Yu Guo [<cyguo@ym.edu.tw>](mailto:%3ccyguo@ym.edu.tw%3e)

**Depends** R (>= 4.0)

**Imports** Rtools, dlnm, splines, foreach, tsModel, Epi

**Description** A function for multivariate analysis of the DLNM

MV.DLNM-function *DLNM that incorporates lag outcomes*

**Examples**

## First, call the MV.DLNM function

## Remember to use the correct the directory where the MV.DLNM code and data are stored

## In this example, the directory is “C:/Users/GUO/Desktop”

## Run MV.DLNM:

MV.DLNM("C:/Users/GUO/Desktop","taipei_data.csv",30)

Note: This example code assumes the maximum lag exposure is 30 day.

Therefore, the R code generates 30 figures with 1 to 30 lag outcomes in the path specified.

**Usage**

MV.DLNM(directory,dataname,maxlagx)

**Arguments**

directory Specify the director for the data.

dataname Name of the data to be analyzed

maxlagx Determine the maximum number of lag exposure
