# Supplementary material for: Extensions of the distributed lag non-linear model (DLNM) to account for cumulative mortality
Source: Environ Sci Pollut Res Int. 2021 Mar 18;28(29):38679–88. doi: 10.1007/s11356-021-13124-0 (PMC8310484; doi:10.1007/s11356-021-13124-0)
Supplement: Supplementary file 3 — (DOCX 18 kb) [file 11356_2021_13124_MOESM3_ESM.docx]

**‘MV.DLNM.SIM’**

**Type** Function

**Title** Simulations for extensions of the Distributed Lag Non-Linear Model (DLNM) to Account for Cumulative Mortality

**Version** 1.0

**Date** Dec 22 2020

**Author** Chao-Yu Guo, Ph.D., Xing-Yi Huang, MS, Pei-Cheng Kuo, MD, Yi-Hau Chen, Ph.D.

**Contact** Dr. Chao-Yu Guo [<cyguo@ym.edu.tw>](mailto:%3ccyguo@ym.edu.tw%3e)

**Depends** R (>= 4.0)

**Imports** Rtools, dlnm, splines, foreach, tsModel, Epi

**Description** A function for multivariate analysis of the DLNM

MV.DLNM.SIM-function *DLNM that incorporates lag outcomes*

**Examples**

## First, call the MV.DLNM.SIM function

## Remember to use the correct the directory where the MV.DLNM.SIM code and data are stored

## In this example, the directory is “C:/Users/GUO/Desktop”

## Run MV.DLNM.SIM:

MV.DLNM.SIM("C:/Users/GUO/Desktop","taipei_data.csv",30,10,1000)

Note: This example code assumes the maximum lag exposure is 30 days, the cumulative lag outcomes are ten days, and 1000 repetitions for simulations. A CSV file named “Final_results.csv” is stored in the path specified after the completion of simulations.

**Usage**

MV.DLNM.SIM(directory,dataname,maxlagx,sumy,repeats)

**Arguments**

directory Specify the director for the data.

dataname Name of the data to be analyzed

maxlagx Determine the maximum number of lag exposure

sumy Determine the number of lag outcome

repeats Determine the number of repetitions for the simulation study
